# The Effectiveness of Curcumin Nanoparticle-Coated Titanium Surfaces in Osteogenesis: A Systematic Review

**DOI:** 10.3390/jfb15090247

**Published:** 2024-08-27

**Authors:** Nandita Suresh, Matti Mauramo, Tuomas Waltimo, Timo Sorsa, Sukumaran Anil

**Affiliations:** 1 Department of Oral and Maxillofacial Diseases, Helsinki University Hospital, Helsinki University, 00014 Helsinki, Finland; nandita.suresh@helsinki.fi (N.S.); tuomas.waltimo@gmail.com (T.W.); timo.sorsa@helsinki.fi (T.S.); 2 Pushpagiri Institute of Medical Sciences and Research Centre, Medicity, Perumthuruthy, Tiruvalla 689101, Kerala, India; 3 Department of Pathology, Helsinki University Hospital, Helsinki University, 00290 Helsinki, Finland; matti.mauramo@helsinki.fi; 4 Faculty of Medicine, University of Basel, 4003 Basel, Switzerland; 5 Department of Oral Diseases, Karolinska Institutet, Huddinge, 171 77 Stockholm, Sweden; 6 Oral Health Institute, Hamad Medical Corporation, Doha P.O. Box 3050, Qatar; 7 College of Dental Medicine, Qatar University, Doha P.O. Box 2713, Qatar

**Keywords:** curcumin nanoparticles, titanium, surface modification, osteogenesis, cell adhesion, cell proliferation, osteogenic differentiation, mineralization, systematic review, in vitro studies

## Abstract

(1) Background: This systematic review critically appraises and synthesizes evidence from in vitro studies investigating the effects of curcumin nanoparticles on titanium surface modification, focusing on cell adhesion, proliferation, osteogenic differentiation, and mineralization. (2) Methods: A comprehensive electronic search was conducted in PubMed, Cochrane Central Register of Controlled Trials, and Google Scholar databases, yielding six in vitro studies that met the inclusion criteria. The search strategy and study selection process followed PRISMA (Preferred Reporting Items for Systematic Reviews and Meta-Analyses) guidelines. A qualitative methodological assessment was performed using the SciRAP (Science in Risk Assessment and Policy) method, which evaluated the reporting and methodological quality of the included studies. (3) Results: All six studies consistently demonstrated that curcumin-coated titanium surfaces inhibited osteoclastogenesis and promoted osteogenic activity, evidenced by enhanced cell adhesion, proliferation, osteogenic differentiation, and mineralization. The mean reporting quality score was 91.8 (SD = 5.7), and the mean methodological quality score was 85.8 (SD = 10.50), as assessed by the SciRAP method. Half of the studies used hydroxyapatite-coated titanium as a control, while the other half used uncoated titanium, introducing potential variability in baseline comparisons. (4) Conclusions: This systematic review provides compelling in vitro evidence supporting the osteogenic potential of curcumin nanoparticle-coated titanium surfaces. The findings suggest that this surface modification strategy may enhance titanium implants’ biocompatibility and osteogenic properties, potentially improving dental and orthopedic implant outcomes. However, the review highlights significant heterogeneity in experimental designs and a concentration of studies from a single research group. Further research, particularly in vivo studies and clinical trials from diverse research teams, is essential to validate these findings and comprehensively understand the translational potential of this promising surface modification approach.

## 1. Introduction

### 1.1. Titanium Implants and Surface Modification

Osseointegration, the direct structural and functional connection between living bone and the surface of a load-bearing implant, stands as a transformative factor in the long-term success of dental and orthopedic implants [1]. This process, first described by Per-Ingvar Brånemark in the 1960s, has not only revolutionized the field of implantology but also inspired the development of stable, long-lasting implants that can effectively restore function and aesthetics [2,3]. The success of osseointegration depends on various factors, including implant material properties, surface characteristics, and the biological response of the surrounding tissues. Titanium and its alloys have been widely used as implant materials due to their excellent biocompatibility, mechanical properties, and corrosion resistance [4]. The natural formation of a stable oxide layer on titanium surfaces contributes to its biocompatibility and resistance to corrosion, making it an ideal material for long-term implantation in the human body [5]. However, despite these advantageous properties, the bioinert nature of titanium surfaces can lead to suboptimal osseointegration, which may result in implant failure, particularly in challenging clinical scenarios or in patients with compromised bone quality [6,7].

The process of osseointegration is a dynamic and complex biological interaction at the bone–implant interface. Initially, proteins from blood and interstitial fluids adsorb onto the implant surface, creating a provisional matrix that influences subsequent cellular responses [8]. Osteoblasts, the bone-forming cells, then attach to this matrix and proliferate and differentiate. As these cells mature, they produce and deposit extracellular matrix proteins, which eventually mineralize to form new bone tissue directly on the implant surface [9]. Concurrently, osteoclasts, bone-resorbing cells, play a crucial role in bone remodeling around the implant, contributing to the dynamic process of osseointegration [10].

Various surface modification strategies have been explored to address the suboptimal osseointegration challenge and enhance titanium implants’ osteogenic properties [7,11]. These approaches aim to alter the implant surface’s physical, chemical, and biological properties to create a more favorable environment for bone formation. Common surface modification techniques include mechanical treatments, chemical treatments, and the application of bioactive coatings [12,13]. Each method aims to enhance osseointegration by improving surface roughness, increasing surface energy, or directly promoting osteoblast adhesion and differentiation.

### 1.2. Curcumin and Its Potential Benefits

In recent years, nanotechnology has emerged as a promising avenue for enhancing implant surface properties. Nanostructured surfaces can mimic the natural extracellular matrix more closely, potentially improving cellular responses and accelerating osseointegration [14,15]. Among the various nanomaterials being investigated, phytonanoparticles have garnered significant attention due to their natural origin and potential bioactive properties [8]. Curcumin nanoparticles, in particular, have emerged as a promising approach to improving titanium surfaces’ biocompatibility and osteogenic potential [16]. Curcumin, a natural polyphenol derived from the rhizome of Curcuma longa (turmeric), has been extensively studied for its diverse therapeutic properties, including potent anti-inflammatory, antioxidant, and osteogenic effects [17]. The traditional use of turmeric in Ayurvedic and Chinese medicine for treating various ailments has sparked scientific interest in harnessing its beneficial properties for modern medical applications [18].

### 1.3. Mechanism of Action of Curcumin on Osteogenesis

Curcumin, the active polyphenolic compound found in turmeric, exerts a multifaceted influence on bone metabolism and osteogenesis through various complex pathways. Its mechanism of action primarily involves upregulating key osteogenic transcription factors, particularly Runx2 and osterix. Curcumin enhances these factors’ nuclear translocation and DNA binding activity, increasing the expression of genes responsible for osteoblast-specific markers and bone matrix proteins [19,20]. Additionally, curcumin stimulates alkaline phosphatase (ALP) activity, a crucial enzyme in bone mineralization. Elevated ALP levels increase inorganic phosphate concentrations, promoting the nucleation and growth of hydroxyapatite crystals, thereby enhancing the deposition and maturation of the mineralized bone matrix [20,21]. Concurrently, curcumin suppresses osteoclastogenesis by inhibiting the RANKL/RANK signaling pathway, which is essential for osteoclast formation and bone resorption [20]. This action helps maintain a favorable balance between bone formation and resorption, promoting net bone accrual. Curcumin’s potent antioxidant and anti-inflammatory properties further contribute to its osteogenic effects by scavenging free radicals and reactive oxygen species, reducing oxidative stress in the bone microenvironment, while also downregulating the production of proinflammatory cytokines such as TNF-α, IL-1β, and IL-6 [22].

Despite these promising effects, the clinical application of curcumin has been hampered by its poor bioavailability, attributed to low water solubility, rapid metabolism, and swift systemic elimination [23]. Researchers have developed innovative approaches to overcome these limitations, such as incorporating curcumin into nanoparticles. This strategy significantly increases its surface area and improves its solubility and stability, potentially enhancing its efficacy in bone regeneration and implant osseointegration [24]. These nanoparticles can be produced using various sophisticated techniques like nanoprecipitation, high-pressure homogenization, and emulsification-solvent evaporation, allowing for controlled size and surface properties that can be tailored to optimize their interaction with biological systems and their release kinetics when applied to implant surfaces [25]. The potential of curcumin in bone regeneration and implant osseointegration is further supported by its ability to promote osteoblast differentiation and function through various mechanisms, including activating key osteogenic transcription factors such as Runx2 and osterix [26]. Additionally, it has demonstrated the ability to inhibit osteoclastogenesis and bone resorption, potentially creating a more favorable balance for net bone formation around implants [27].

Furthermore, researchers have further explored combining curcumin with bioenhancers to improve its bioavailability. For instance, piperine, a compound found in black pepper, has been shown to inhibit the enzymes responsible for metabolizing curcumin, thereby enhancing its absorption and bioavailability [28]. This synergistic approach demonstrates the potential for combining natural compounds to overcome inherent limitations and enhance therapeutic efficacy. Advanced formulations have also been developed to address curcumin’s bioavailability issues. Liposomal curcumin, which encapsulates curcumin in lipid bilayers, and curcumin phytosomes, which bind curcumin to phospholipids, have shown promise in enhancing cellular uptake and prolonging circulation time [29,30]. These sophisticated delivery systems ensure that curcumin can exert its therapeutic effects more effectively in the body, potentially leading to improved outcomes when applied to implant surfaces. By incorporating curcumin nanoparticles onto titanium surfaces, researchers aim to harness these beneficial effects to promote bone formation and accelerate osseointegration [9,31]. The nanoparticle formulation not only addresses the bioavailability issues of curcumin but also allows for controlled release at the implant site, potentially providing sustained beneficial effects throughout the critical early stages of osseointegration.

### 1.4. Rationale for the Review

Several in vitro studies have explored the promising effects of curcumin nanoparticle-modified titanium surfaces on various aspects of osteogenesis, including cell adhesion, proliferation, differentiation, and mineralization [9,15,18,31,32,33]. These studies have employed a variety of cell types, such as mesenchymal stem cells, osteoblasts, and osteoclasts, to evaluate the cellular response to curcumin nanoparticle-modified surfaces [34]. The use of different cell types offers a comprehensive understanding of how curcumin nanoparticles might influence the intricate cellular interactions involved in osseointegration. Despite the growing body of research in this area, a comprehensive synthesis of the available evidence is necessary to draw definitive conclusions about the efficacy of this surface modification approach. The heterogeneity in experimental designs, cell types used, and outcome measures across studies makes it challenging to understand the true potential of curcumin nanoparticle-modified titanium surfaces. Moreover, the scarcity of in vivo and clinical studies assessing the osteogenicity of curcumin-modified titanium implants highlights a significant gap in the current research landscape. While in vitro studies provide valuable insights into cellular mechanisms and potential effects, they cannot fully replicate the complex biological environment encountered by implants in living organisms. Translating promising in vitro results to clinically relevant outcomes remains critical in developing new implant technologies.

This systematic review aims to critically appraise and synthesize current in vitro evidence on the effects of curcumin nanoparticle-modified titanium surfaces on osteogenesis. We will examine methods for creating these modified surfaces and their impact on cell adhesion, proliferation, viability, and osteogenic differentiation. The review will assess the effects on mineralization, extracellular matrix formation, and osteoclast activity and explore potential mechanisms of action. By analyzing these aspects across multiple studies, we seek to identify trends, discrepancies, and areas needing further investigation. Our findings will provide an overview of the evidence supporting curcumin nanoparticles in titanium surface modification, highlight gaps in current literature, and inform future research directions. This review’s findings are crucial for developing novel surface modification strategies, which could revolutionize the field of osseointegration and significantly improve outcomes for dental and orthopedic implant patients.

## 2. Materials and Methods

### 2.1. Protocol and Registration

**Figure 1:**
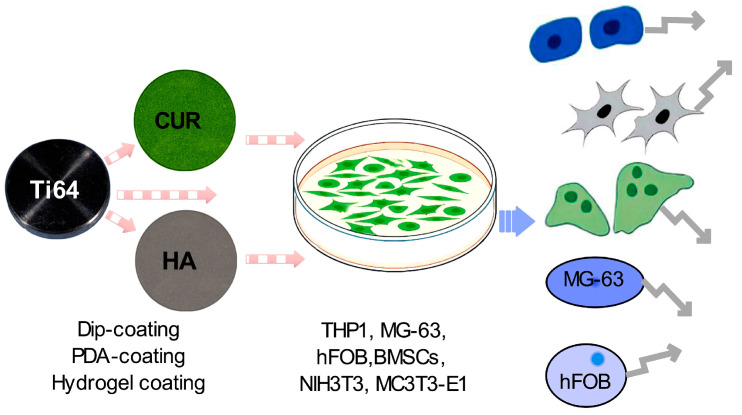
Schematic representation of a curcumin-enhanced titanium implant coating experiment. The diagram illustrates the potential influence of the coated implant on various cellular processes relevant to bone regeneration and implant integration. Ti64 alloy is coated with curcumin (CUR) and hydroxyapatite (HA) using various coating methods. Cell culture dish containing multiple cell types (THP1, MG-63, hFOB, BMSCs, NIH3T3, and MC3T3-E1). Hypothesized effects on different cell types, including osteoblasts, osteoclasts, immune cells, MG-63, and hFOB cells, as indicated by arrows.

Protocol and Registration: This systematic review followed the Preferred Reporting Items for Systematic Reviews and Meta-analyses (PRISMA) guidelines (Figure 1). The protocol was registered in INPLASY with registration number—INPLASY202470105 and https://doi.org/10.37766/inplasy2024.7.0105 (accessed on 26 July 2024).

### 2.2. Eligibility Criteria

We employed the PICOS (Population, Intervention, Comparison, Outcome, Study Design) framework to define the eligibility criteria for this systematic review. The population consisted of in vitro studies analyzing osteogenicity after incorporating curcumin on titanium surfaces. The intervention of interest was curcumin nanoparticle-modified titanium discs or surfaces. For comparison, we considered uncoated titanium discs, hydroxyapatite-coated titanium discs, and other relevant surface modifications. Primary outcomes included cell adhesion, proliferation, osteogenic differentiation, and mineralization, while secondary outcomes encompassed gene expression of osteogenic markers, protein production, and effects on osteoclastogenesis. Regarding study design, we included original research articles published in peer-reviewed journals in English.

### 2.3. Focused Question

The primary research question guiding this review was: “What is the effect of curcumin nanoparticles coated on titanium surfaces in enhancing osteogenesis in vitro, focusing on cell adhesion, proliferation, osteogenic differentiation, and mineralization?”

### 2.4. Inclusion and Exclusion Criteria

The inclusion criteria for this systematic review were as follows: We included original research articles published in English that were in vitro studies investigating the effects of curcumin nanoparticles on titanium surface modification. To be eligible, studies had to report at least one primary outcome, including cell adhesion, proliferation, osteogenic differentiation, or mineralization. Additionally, we included studies that used relevant cell types such as osteoblasts, mesenchymal stem cells, or osteoclasts. For the exclusion criteria, we eliminated several types of publications from consideration. Review articles, case reports, conference abstracts, editorials, and opinion pieces were excluded. We also excluded in vivo or clinical studies, as our focus was specifically on in vitro research. Studies that did not involve curcumin nanoparticles or titanium surface modification were not considered. Similarly, we excluded studies focusing solely on curcumin’s effects without titanium surface modification, as the interaction between curcumin nanoparticles and titanium surfaces was central to our research question.

### 2.5. Information Sources and Search Strategy

A comprehensive electronic search was performed using PubMed, Cochrane Central Register of Controlled Trials, and Google Scholar databases. The search covered the period from the inception of each database up to March 2024, with no lower date limit. The search strategy included combinations of controlled vocabulary (MeSH terms) and free-text terms related to curcumin nanoparticles, titanium, surface modification, and osteogenesis.

The following search string was used for PubMed, with appropriate modifications for other databases:
((curcumin[MeSH Terms]) OR (curcumin*[Title/Abstract]) OR (diferuloylmethane[Title/Abstract])) AND ((nanoparticle*[Title/Abstract]) OR (nano*[Title/Abstract])) AND ((titanium[MeSH Terms]) OR (titanium*[Title/Abstract])) AND ((surface modification[MeSH Terms]) OR (surface modif*[Title/Abstract]) OR (coating*[Title/Abstract])) AND ((osteogenesis[MeSH Terms]) OR (osteogen*[Title/Abstract]) OR (osseointegration[Title/Abstract]) OR (bone formation[Title/Abstract]))

We manually searched the reference lists of included studies and relevant review articles to identify any additional eligible studies.

### 2.6. Study Selection

Two reviewers (AS and NS) independently screened the titles and abstracts of all identified studies using the predetermined eligibility criteria. The same two reviewers retrieved and independently evaluated the full texts of potentially eligible studies. Any disagreements at either stage were resolved through discussion and consensus, with a third reviewer (TW) consulted when necessary.

The selection process was documented using a PRISMA flow diagram, detailing the number of studies identified, screened, assessed for eligibility, and included in the final review, along with reasons for exclusions at each stage.

### 2.7. Data Collection Process and Data Items

Two reviewers (AS and NS) independently extracted data using a pre-designed, standardized Microsoft Excel spreadsheet. From each included study, they extracted a comprehensive set of data items. These included study characteristics such as authors, year of publication, and country of origin. Information about the titanium substrate was collected, including the type of titanium and surface preparation methods. The reviewers also extracted details about the curcumin nanoparticles, noting their concentration, size, and preparation method. The surface modification technique used to incorporate curcumin nanoparticles onto titanium surfaces was recorded, as were the cell types used in each study.

The experimental design of each study was documented, including details about the groups, sample sizes, and duration of experiments. A wide range of outcomes was extracted, encompassing cell adhesion measures, cell proliferation assays and results, osteogenic differentiation markers and assays, mineralization assessments, gene expression analyses, protein production measurements, and effects on osteoclastogenesis (where assessed). Finally, the key findings and conclusions of each study were noted.

To ensure accuracy and consistency, any discrepancies in data extraction were resolved through discussion between the two reviewers. In cases where agreement could not be reached, a third reviewer (TW) was consulted to make the final decision. This rigorous process ensured that all relevant data were accurately captured for subsequent analysis and synthesis.

### 2.8. Risk of Bias in Individual Studies

The quality assessment of the included studies was performed using the Science in Risk Assessment and Policy (SciRAP) method [35]. This tool was selected for its comprehensive evaluation of relevance, reporting quality, and methodological quality in toxicological and ecotoxicological studies, aligning well with the in vitro nature of the studies in this review. Two reviewers (AS and NS) independently assessed each study using the SciRAP tool. The assessment covered three main domains: reporting quality, methodological quality, and relevance. The reporting quality domain evaluated the completeness and clarity of reported information. The methodological quality assessment focused on the reliability and validity of the study methods. The relevance domain determined the applicability of the study to the research question.

Each domain was scored, and each study’s overall quality score was calculated. Any disagreements between the two reviewers were resolved through discussion to ensure consistency and accuracy in the quality assessment process. In cases where consensus could not be reached, a third reviewer (TW) was consulted to make the final determination. This rigorous approach to quality assessment provided a comprehensive evaluation of the risk of bias in the included studies, enhancing the reliability of the review’s findings.

### 2.9. Summary Measures and Synthesis of Results

Due to the anticipated heterogeneity in study designs, cell types, and outcome measures, a meta-analysis was deemed not feasible for this review. Instead, we conducted a qualitative synthesis of the included studies, focusing on the effects of curcumin nanoparticle-modified titanium surfaces on cell adhesion, proliferation, osteogenic differentiation, and mineralization. The synthesis was structured around several key aspects to provide a comprehensive overview of the current evidence. We examined the methods of incorporating curcumin nanoparticles onto titanium surfaces and their effects on cell adhesion and initial attachment. The impact on cell proliferation and viability was also assessed. We analyzed the influence of these modified surfaces on osteogenic differentiation, including changes in gene expression and protein production of key osteogenic markers. The effects on mineralization and the formation of calcified extracellular matrix were evaluated. Additionally, we considered any observed effects on osteoclast formation or activity. Finally, we explored the proposed mechanisms of action for curcumin nanoparticles in enhancing osteogenesis.

To further elucidate potential differences in outcomes, we conducted a subgroup analysis based on the type of control used, comparing studies that used uncoated titanium versus those that used hydroxyapatite-coated titanium as controls. The results of our analysis were narratively summarized. In our synthesis, we also highlighted any inconsistencies or contradictions in the results across different studies and discussed potential reasons for these discrepancies. This approach allowed us to provide a nuanced understanding of the current state of research on curcumin nanoparticle-modified titanium surfaces and their potential for enhancing osteogenesis.

### 2.10. Additional Analyses

To address the concern about the concentration of studies from a single research group, we conducted a sensitivity analysis excluding these studies to determine if the overall conclusions of the review would change significantly. We also analyzed the included studies chronologically to identify trends or evolutions in methodologies or findings over time. By implementing these detailed methods, we aimed to provide a comprehensive and transparent review of the current in vitro evidence on the effects of curcumin nanoparticle-modified titanium surfaces on osteogenesis while addressing the limitations and potential biases in the existing literature.

## 3. Results

**Figure 2:**
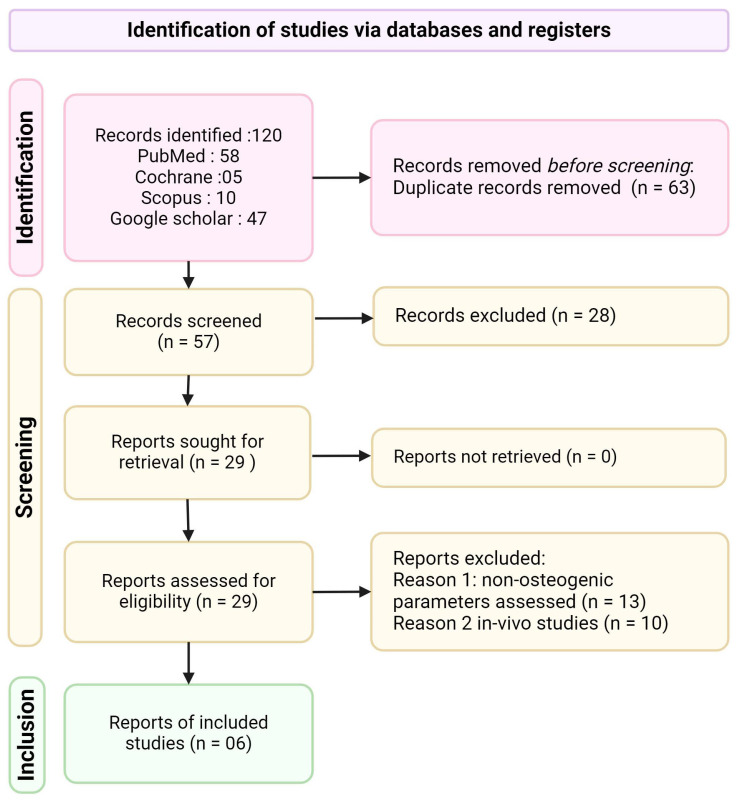
PRISMA flow diagram of the study selection process.

The initial database search yielded 120 articles. After removing 63 duplicates, 57 articles were screened for eligibility. Following title and abstract screening, 29 articles underwent full-text assessment. Ultimately, six articles met the inclusion criteria and were included in this systematic review (Figure 2). The six included studies were published between 2015 and 2024, indicating a growing interest in this field. Three of the six studies were conducted by the same research group, which is an important consideration when interpreting the results. Table 1 provides a comprehensive overview of the study characteristics, including authors, year of publication, titanium substrate characteristics, curcumin nanoparticle preparation methods, surface modification techniques, cell types used, control samples, and key outcome measures.

### Study Characteristics

The quality assessment of the included studies was conducted using the SciRAP method (Table 2). The mean reporting quality score was 91.8 (SD = 5.7), with all studies describing the test sample (curcumin-coated titanium) in detail. However, only one study adequately described the control, including the positive control. The mean methodological quality score was 85.8 (SD = 10.50). All studies performed statistical evaluations, but one only partially fulfilled the criteria. Notably, no studies have described allocation concealment or assessment through blinding. The consistently high scores across studies raise questions about the sensitivity of the SciRAP tool in detecting methodological issues in this specific type of research.

Table 3 summarizes the surface modification techniques and curcumin concentrations used in the included studies. The most common methods for incorporating curcumin nanoparticles onto titanium surfaces were dip-coating [15,18,31], polydopamine-mediated coating [9,32], and hydrogel coating [33]. Curcumin concentrations varied across studies, ranging from 10 µM to 500 µg in ethanol solutions. Studies also incorporated additional bioactive molecules such as Vitamin K, EGCG, and Acemannan [15,33].

The cell types and outcome measures used in each study are outlined in Table 4. The most frequently used cell lines were the human fetal osteoblast (hFOB), the human osteosarcoma cell line (MG-63), and bone marrow mesenchymal stem cells (BMSCs). Primary outcome measures consistently included cell viability assays (MTT or CCK8) and alkaline phosphatase (ALP) activity. Secondary outcome measures varied but often included gene expression analyses, protein production measurements, and assessments of mineralization.

Key findings from the studies revealed consistent trends in the effects of curcumin nanoparticle-coated titanium surfaces on various aspects of osteogenesis. All studies reported increased cell viability and proliferation of osteoblasts (hFOB) and BMSCs on curcumin-coated surfaces compared to controls. Interestingly, three studies reported the inhibition of MG-63 (osteosarcoma) cell viability on curcumin-coated titanium, suggesting a potential selective action. This differential effect on normal bone-forming cells versus cancer cells warrants further investigation. Regarding osteogenic differentiation, increased ALP activity was reported in four studies, and enhanced mineralization, as evidenced by Alizarin Red staining, was observed in three studies. One study reported the upregulation of osteogenic genes (e.g., OCN, ALP, and COL-1), further supporting the osteogenic potential of curcumin-coated surfaces. Three studies also reported inhibition of osteoclastogenesis, as evidenced by reduced TRAP-positive cell formation and decreased expression of osteoclast-related genes (RANK, RANKL). Concentration-dependent effects were noted, which found that curcumin concentrations of 10 µM and 20 µM had the highest cell viability of BMSCs, suggesting an optimal concentration range for osteogenic effects [9,32]. This finding highlights the importance of dose optimization in future studies and potential clinical applications. Three studies investigated the effects of curcumin in combination with other bioactive molecules (Vitamin C, Vitamin K, and EGCG), showing enhanced osteogenic effects compared to curcumin alone. These synergistic effects suggest potential avenues for developing more potent surface modification strategies. While these results consistently demonstrate the potential of curcumin nanoparticle-coated titanium surfaces in enhancing osteogenesis and inhibiting osteoclastogenesis, the heterogeneity in study designs, cell types, and outcome measures necessitates cautious interpretation of these findings. Future studies should aim to standardize protocols and expand on these promising initial results.

## 4. Discussion

This systematic review aimed to assess the osteogenic potential of curcumin nanoparticle-coated titanium surfaces by analyzing in vitro studies published up to March 2024. The findings from the six included studies consistently demonstrate the beneficial effects of curcumin nanoparticles on titanium surfaces in enhancing osteogenesis and osseointegration. However, the limited number of studies and their heterogeneity necessitate a cautious interpretation of these results.

### 4.1. Summary of Main Findings

The systematic review of curcumin nanoparticle-coated titanium surfaces revealed several significant effects on osteogenesis. Consistently across studies, enhanced osteoblast and mesenchymal stem cell (MSC) viability and proliferation were observed [15,31,32], indicating improved biocompatibility and cellular response to the modified surfaces. This was accompanied by increased expression of key osteogenic genes, including RUNX2, ALPL, OCN, and COL-1 [32,36], suggesting a direct stimulatory effect on osteogenic differentiation pathways. Notably, curcumin nanoparticles also inhibited osteoclastogenesis [15,18], potentially creating a more favorable balance for net bone formation. Synergistic effects were observed when curcumin was combined with other bioactive molecules such as Vitamin K and epigallocatechin gallate (EGCG) [15,18,31], indicating the potential for developing more potent surface modification strategies. Furthermore, immunomodulatory effects through macrophage polarization were reported [36], suggesting that curcumin nanoparticles may influence the local immune environment to promote osteogenesis. These multifaceted effects highlight the potential of curcumin nanoparticle coatings to enhance the osteogenic properties of titanium implants through various complementary mechanisms.

### 4.2. Mechanisms of Action

The mechanisms underlying curcumin nanoparticles’ osteogenic effects on titanium surfaces appear multifaceted and synergistic. At the molecular level, curcumin enhances osteoblastic cell proliferation by upregulating key osteogenic genes such as RUNX2 and ALPL [32,37,38,39,40]. Concurrently, curcumin exhibits an inhibitory effect on osteoclastogenesis by interfering with RANK-L signaling and monocyte differentiation [41], a finding consistently reported across multiple studies [27,41,42]. This dual action on bone-forming and bone-resorbing cells creates a favorable balance for net bone formation. Furthermore, curcumin’s immunomodulatory properties, particularly its ability to induce polar differentiation of macrophages, have been demonstrated to contribute to an osteogenic microenvironment [32,43]. The inherent antioxidant and anti-inflammatory properties of curcumin’s phytochemical components, including anthocyanidins, flavonoids, and volatile oils [24,44], likely play a supportive role in creating an optimal environment for bone formation. Curcumin is selective, promoting osteoblast and MSC activity while inhibiting osteosarcoma cells [34,45]. This selectivity enhances its potential for improving osseointegration and suggests possible applications in preventing or treating bone-related malignancies. Collectively, these diverse mechanisms of action underscore the potential of curcumin nanoparticle coatings to significantly enhance the osteogenic properties of titanium implants through a comprehensive and multitargeted approach.

### 4.3. Critical Analysis of Study Designs and Outcomes

While the reviewed studies consistently demonstrate the positive effects of curcumin nanoparticles on osteogenesis, several critical factors merit careful consideration when interpreting these results. The heterogeneity in experimental designs, including variations in titanium substrates (HA-coated versus uncoated), cell types, and outcome measures, presents a significant challenge in drawing direct comparisons between studies. This diversity underscores the need for more standardized protocols in future research to facilitate meta-analyses and more robust conclusions. The concentration-dependent effects of curcumin, as reported by Chen et al. [32], highlight the importance of establishing clear dose–response relationships across different cell types and experimental conditions. Moreover, the promising synergistic effects observed when combining curcumin with other bioactive molecules such as Vitamin C, Vitamin K, and EGCG warrant further investigation to develop more effective surface modification strategies [15,18,31]. However, it is crucial to note that the included studies primarily focused on short-term in vitro effects, leaving unanswered questions about the long-term stability of curcumin nanoparticle coatings and their sustained effects under physiological conditions. Perhaps most importantly, while the in vitro results are encouraging, the translation to in vivo conditions remains a significant hurdle. The complex in vivo environment, with factors such as protein adsorption, immune responses, and mechanical stresses, could significantly influence the performance of curcumin-coated implants. These critical considerations underscore the need for a cautious interpretation of the current findings and highlight important directions for future research in this promising field.

### 4.4. Limitations of the Current Review

While providing valuable insights, this systematic review has several important limitations to consider when interpreting its findings. Foremost among these is the limited number of studies meeting the inclusion criteria, with only six studies ultimately analyzed. This small sample size inherently restricts the generalizability of the findings and underscores the nascent nature of research in this field. Compounding this limitation is the concentration of studies from a single research group, with half of the included studies conducted by the same team. This potentially introduces bias and limits the diversity of experimental approaches represented in the review. Furthermore, the lack of standardization in control samples across studies, with some using HA-coated titanium and others using uncoated titanium, complicates direct comparisons and makes it challenging to draw definitive conclusions about the relative effectiveness of curcumin nanoparticle coatings. The consistently positive results reported across all included studies raise concerns about potential publication bias, suggesting that negative or null findings may be underrepresented in the literature. Lastly, the limitations of the SciRAP tool used for quality assessment became apparent, as the high-quality scores across studies suggest it may not be sufficiently sensitive to detect methodological issues specific to this type of biomaterials research. These limitations collectively highlight the need for a cautious interpretation of the review’s findings and underscore the importance of further, more diverse research in this promising area of implant surface modification.

### 4.5. Future Research Directions

Several key research directions emerge as priorities to address the limitations identified in this review and advance the field of curcumin nanoparticle-coated titanium surfaces. Firstly, developing standardized protocols for curcumin nanoparticle preparation, titanium surface modification, and outcome assessments is crucial to facilitate meaningful study comparisons. Comprehensive dose–response studies are needed to identify optimal curcumin concentrations for different cell types and applications, addressing the current gap in our understanding of concentration-dependent effects. Long-term stability assessments under simulated physiological conditions are essential to predict the durability of these coatings in vivo. Deeper mechanistic studies should elucidate the cellular and molecular pathways underlying curcumin’s osteogenic and osseointegration properties, including its effects on signaling cascades and epigenetic regulation. The field would greatly benefit from well-designed in vivo studies to evaluate the performance of curcumin nanoparticle-coated implants under true physiological conditions and comparative studies directly assessing their efficacy against other established surface modification techniques. Ultimately, clinical trials with diverse patient populations are necessary to test the real-world applicability of these implants in orthopedics and dentistry. Additionally, exploring alternative quality assessment tools that are more sensitive to methodological issues in biomaterials research could enhance the rigor of future systematic reviews in this field. While the evidence suggests promising osteogenic potential for curcumin nanoparticle-coated titanium surfaces, these proposed research directions are critical to establishing their efficacy and safety for clinical applications. As this research progresses, curcumin nanoparticle coatings may emerge as a valuable strategy for enhancing the performance of titanium implants, potentially transforming outcomes in orthopedic and dental applications.

## 5. Conclusions

This systematic review of in vitro studies provides promising evidence supporting the osteogenic potential of curcumin nanoparticle-coated titanium surfaces. The included studies consistently demonstrated that curcumin nanoparticles promoted cell adhesion, proliferation, osteogenic differentiation, and mineralization while inhibiting osteoclastogenesis. However, several important limitations and considerations must be acknowledged when interpreting these results.

## Figures and Tables

**Table 1:** Characteristics and key findings of included studies on curcumin nanoparticle-coated titanium surfaces.

Authors, Year	Titanium Substrate	Curcumin Preparation	Surface Modification Method	Cell Types	Control Sample	Key Outcome Measures	Main Findings
Majumdar & Bose, 2024 [15]	HA-coated Ti64 alloy, 12.5 × 2 mm	500 μg CUR in ethanol (1:4 *w*/*v* ratio)	Dip-coating	hFOB, THP1 monocytes, MG-63	HA-coated Ti	MTT assay, TRAP assay	CUR coating increased osteogenic potential and inhibited osteoclast activity. Synergistic effect with Vitamin K.
Kushram et al., 2023 [31]	HA-coated Ti64 alloy, 12.5 × 2 mm	500 μg CUR in ethanol (1:4 *w*/*v* ratio)	Dip-coating	MG-63, hFOB	HA-coated Ti	MTT assay, ALP assay, Immunohistochemistry	CUR inhibited MG-63 growth and promoted hFOB viability. Synergistic effect with EGCG.
Chen et al., 2022 [32]	Ti-5 wt% Cu alloy, 10 × 2 mm	Aqueous CUR 1 mg/mL, 10 μM and 20 μM	Polydopamine-mediated coating	BMSCs	Uncoated Ti-Cu alloy	CCK8 assay, ALP activity, Gene expression	10 μM and 20 μM CUR enhanced BMSC osteogenic differentiation through macrophage regulation.
Sarkar & Bose, 2020 [18]	HA-coated Ti6Al4V Grade 5, 12.2 × 2 mm	0.5 g CUR in ethanol	Dip-coating	hFOB, MG-63	HA-coated Ti	MTT assay, Release kinetics	CUR showed pH-dependent release, inhibited osteoclasts at pH 5.2, and promoted osteogenesis at pH 7.4.
Chowdhary, 2018 [33]	Grade IV Ti, 5 × 2 mm	25%, 50%, 75% CUR with acemannan	Hydrogel coating	MG-63	Uncoated Ti	MTT assay, ALP activity	CUR-hydrogel inhibited MG-63 cells. 10 μM and 20 μM CUR safe for osteoblasts.
He et al., 2015 [9]	Ti Sheet 1 × 1 cm	0.1 mL CUR (10 μM and 20 μM) in water	Polydopamine-mediated coating	NIH3T3, MC3T3-E1	Uncoated Ti	MTT assay, ALP activity, Ca deposition	10 μM and 20 μM CUR did not adversely affect osteoblast viability or proliferation.

**Table 2:** Reporting and methodological quality scores using the SciRAP tool.

Study/Year	Reporting Quality Score	Methodological Quality Score	Key Strengths	Key Limitations
Majumdar et al., 2024 [15]	86.84	73.08	Detailed description of test samples	Inadequate control description
Khushram et al., 2023 [31]	94.74	84.62	Comprehensive statistical evaluation	Lack of blinding in assessments
Chen D et al., 2022 [32]	94.74	96.15	Robust methodological approach	Minor reporting inconsistencies
Sarkar et al., 2020 [18]	97.37	84.62	Excellent reporting quality	Some methodological details unclear
Chowdhary et al., 2018 [33]	82.5	76.92	Clear outcome measures	Incomplete statistical analysis
He et al., 2015 [9]	94.74	100	Exemplary methodological quality	Minor reporting omissions
**Mean**	**91.82**	**85.** **90**	-	-
**Standard Deviation**	**5.79**	**10.50**	-	-

**Table 3:** Surface modification techniques and curcumin concentrations.

Study	Surface Modification Technique	Curcumin Concentration	Substrate Type	Additional Bioactive Molecules
Majumdar et al., 2024 [15]	Dip-coating	500 μg in ethanol (1:4 *w*/*v* ratio)	HA-coated Ti64 alloy	Vitamin K
Khushram et al., 2023 [31]	Dip-coating	500 μg in ethanol (1:4 *w*/*v* ratio)	HA-coated Ti64 alloy	EGCG
Chen D et al., 2022 [32]	Polydopamine-mediated coating	10 μM and 20 μM in aqueous solution	Ti-5 wt% Cu alloy	None
Sarkar et al., 2020 [18]	Dip-coating	0.5 g in ethanol	HA-coated Ti6Al4V Grade 5	Vitamin K2
Chowdhary et al., 2018 [33]	Hydrogel coating	25%, 50%, 75% with acemannan	Grade IV Ti	Acemannan
He et al., 2015 [9]	Polydopamine-mediated coating	10 μM and 20 μM in water	Ti Sheet	None

**Table 4:** Cell types and outcome measures.

Study	Cell Types	Primary Outcome Measures	Secondary Outcome Measures
Majumdar et al., 2024 [15]	hFOB, THP1 monocytes, MG-63	MTT cell viability assay	TRAP assay for osteoclast activity
Khushram et al., 2023 [31]	MG-63, hFOB	MTT assay, ALP assay	Immunohistochemistry
Chen D et al., 2022 [32]	BMSCs	CCK8 assay for cell viability	ALP activity, Osteogenic gene expression
Sarkar et al., 2020 [18]	hFOB, MG-63	MTT Cell Viability Assay	pH-dependent release kinetics
Chowdhary et al., 2018 [33]	MG-63	MTT assay	Alkaline phosphatase activity assay
He et al., 2015 [9]	NIH3T3, MC3T3-E1	MTT assay	ALP activity, Calcium deposition

## Data Availability

The original contributions presented in the study are included in the article, further inquiries can be directed to the corresponding author.
